# Correction: Loss of tuberous sclerosis complex 2 sensitizes tumors to nelfinavir–bortezomib therapy to intensify endoplasmic reticulum stress-induced cell death

**DOI:** 10.1038/s41388-018-0598-0

**Published:** 2019-01-08

**Authors:** Charlotte E. Johnson, Elaine A. Dunlop, Sara Seifan, Henry D. McCann, Trevor Hay, Geraint J. Parfitt, Ashley T. Jones, Peter J. Giles, Ming H. Shen, Julian R. Sampson, Rachel J. Errington, D. Mark Davies, Andrew R. Tee

**Affiliations:** 1 Division of Cancer and Genetics, Cardiff University, Heath Park, Cardiff, CF14 4XN UK; 2 European Cancer Stem Cell Research Institute, Cardiff University, Hadyn Ellis Building, Maindy Road, Cardiff, CF24 4HQ UK; 3 Department of Oncology, South West Wales Cancer Centre, Singleton Hospital, Swansea, SA2 8QA UK

**Correction to: Oncogene**

10.1038/s41388-018-0381-2

This article was originally published under standard licence, but has now been made available under a CC BY 4.0 license. The PDF and HTML versions of the paper have been modified accordingly.

